# *Brugia malayi* microfilariae transport alphaviruses across the mosquito midgut

**DOI:** 10.1371/journal.pone.0172309

**Published:** 2017-02-21

**Authors:** Jefferson A. Vaughan, Michael J. Turell

**Affiliations:** Virology Division, U.S. Army Medical Research Institute for Infectious Diseases, Fort Detrick, Maryland, United States of America; University of California Davis, UNITED STATES

## Abstract

Concurrent ingestion of microfilariae (MF) and arboviruses by mosquitoes can enhance mosquito transmission of virus compared to when virus is ingested alone. Within hours of being ingested, MF penetrate the mosquito midgut and introduce virus into mosquito hemocoel, creating a disseminated viral infection much sooner than normal. How virus is actually introduced is not known. In this report, we present experimental evidence that suggests that certain alphaviruses may adhere or otherwise associate with sheathed *Brugia malayi* MF in the blood of a dually-infected host and that the virus is carried into the mosquito hemocoel by the MF during their penetration of the mosquito midgut. The mechanism of MF enhancement may be more complex than simple leakage of viremic blood into the hemocoel during MF penetration. The affinity of arboviruses to adhere to or otherwise associate with MF may depend on the specific combination of the virus and MF involved in a dual host infection. This in turn may determine the relative importance that MF enhancement has within an arbovirus transmission system.

## Introduction

The basic mechanism of arboviral infection in mosquitoes has been known for many years [1]. Briefly, ingested virus enters and replicates within the mosquito midgut epithelium cells, disseminates into the hemocoel, and subsequently infects the mosquito salivary glands to be excreted together with saliva during blood feeding. But not every mosquito species is capable of transmitting every arboviral species. For most mosquito-virus combinations, there are barriers to the infection process. The most important barriers to viral infectivity are the "midgut infection" and “midgut escape" barriers [2,3]. In many cases once these midgut barriers are overcome, viral infection of the salivary glands ensues. Thus, any mechanism that allows virus to bypass the midgut will greatly increase the potential transmission of arboviruses by mosquitoes.

**Fig 1:**
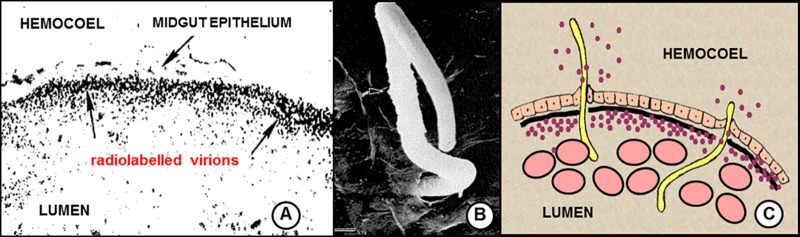
Conventionally-accepted mechanism of microfilarial enhancement of arboviral transmission. Blood meal coagulation in the midgut of mosquitoes fed on viremic live host causes concentration of the ingested virions to the periphery of the midgut lumen. **A**. Transverse close-up of engorged *Culex tarsalis* mosquito midgut showing the spatial distribution of ingested radiolabeled western equine encephalitis virions 30 minutes after feeding on a viremic chick. Note the band of concentrated virions (arrows) accumulated underneath midgut epithelium after mosquitoes fed on viremic host. From Weaver *et al*. 1993. [11]. **B**. Electron micrograph showing penetration and emergence of *Brugia pahangi* mf from *Aedes aegypti* midgut (note emergence hole). From Christensen & Sutherland 1984 [12]. **C.** Composite diagram illustrating conventionally accepted mechanism of microfilarial enhancement. As microfilariae traverse the midgut, some of the virions within the lumen leak into the hemocoel from the exit hole.

In nature, vertebrate hosts of arboviruses are frequently infected with filarial nematodes. As part of their life cycle, microfilariae (MF) penetrate the mosquito midgut after being ingested and move to their preferred site of development within the hemocoel (e.g., flight muscle, fat body, *etc*.). Laboratory studies have shown that MF in the blood can enhance the infectivity of arboviruses to mosquitoes by enabling virus to bypass the midgut barriers [4–9]. If the blood meal also contains virus, some of the virus can enter the hemocoel (see Fig 1), circumventing the initial developmental events required for normal arboviral infection of mosquitoes (i.e., viral attachment, invasion, replication and release from within the mosquito midgut epithelium). This phenomenon has been termed ***microfilarial enhancement of arboviral transmission*** [8,9] and has been shown experimentally to alter two important transmission parameters for arboviruses. First, microfilarial enhancement can transform poorly susceptible mosquitoes with midgut barriers into fully susceptible mosquitoes (i.e., increase vector competence). This can increase the number of secondary vectors involved in an arbovirus transmission cycle. Second, MF enhancement can simultaneously shorten the time interval between when a mosquito feeds on a viremic host and when the mosquito becomes able to transmit virus by bite (i.e. decrease extrinsic incubation period). This has the potential to increase arbovirus transmission dramatically. Indeed, computer simulations on the effect of human Brugian filariasis on dengue epidemics [10] indicated that the accelerating effect of MF enhancement on dengue virus development within the vector, *Aedes aegypti*, would produce not only a higher-than-normal incidence of dengue during the initial year of its introduction, but would also generate more frequent (although not necessarily larger) epidemic waves over a 14-year simulation period.

The mechanism of virus introduction into the hemocoel is not understood but generally assumed to result from the leakage of viremic blood through midgut punctures made by MF (Fig 1). If a sufficient quantity of virus seeps into the hemocoel during MF penetration, the mosquito will develop a disseminated infection and MF enhancement will occur. In our early study with eastern equine encephalitis virus (EEEV) and *Brugia malayi* MF [8], rates of viral dissemination were compared among mosquitoes fed on gerbils with dual infections (MF + virus) versus gerbils with single infections (virus only). Three different species of *Aedes* mosquitoes were fed concurrently on these animals but in those fed on the dually-infected gerbils, different levels of enhanced viral dissemination resulted, despite the fact that the three mosquito species were shown to be equally susceptible to inoculated EEEV. Microfilarial enhancement was pronounced in one species (*Ae*. *taeniorhynchus*), moderate in another (*Ae*. *aegypti*) and absent altogether in a third (*Ae*. *triseriatus*). These differences were attributed to differences in the quantity of blood that leaked into the hemocoel during MF penetration. However when a different virus—Venezuelan equine encephalitis virus (VEEV)–was tested in the same *Aedes* / *B*. *malayi* system but in gerbils that had both lower viremias (2- to 10-fold) and lower microfilaremias (ca. 2-fold) [9], the magnitude of enhanced dissemination was equal to or higher than that observed with EEEV. The most parsimonious explanation for these incongruous results was that the mosquitoes were simply more susceptible to VEEV than to EEEV and therefore it took less VEEV leaking into the hemocoel to achieve the same level of enhancement as with EEEV. But that explanation was rejected after we analyzed the results of intrathoracic inoculations with both viruses and found that the mosquitoes were actually *less* susceptible to VEEV (i.e., higher ID-50 values) than to EEEV (Table 1, S1 Table). Clearly, blood meal leakage was not the complete story. In this report, we provide three lines of evidence to suggest that, in addition to passive leakage of viremic blood into the hemocoel, virus can also be actively transported across the midgut by MF. Furthermore, the degree to which this ‘co-transport’ occurs depends on the specific combination of MF and virus species involved. The lines of evidence include; 1) quantitative estimates of viremic blood introduced per MF, 2) differential kinetics of virus growth within the hemocoels of orally-infected versus inoculated mosquitoes, and 3) serial dilution ‘spin-and-wash’ experiments.

**Table 1:** Theoretical amount of viremic blood, expressed as parts per blood meal (ppBM x 10−^7^) that would have to be introduced into the hemocoel per penetrating *Brugia malayi* microfilaria (MF) in order to result in the level of enhanced viral dissemination reported by Vaughan and Turell 1996 [8], Vaughan et al. 1999 [9].

Mosquito species	Virus	Innate susceptibility (ID-50) *	Trial No.	Amount of virus ingested per mosquito (PFU)	Geometric mean number of MF penetrating the midgut	Net enhancement in viral dissemination	ppBM x 10−^7^ per MF
*Ae*. *taeniorhynchus*	VEEV	0.20	1	250	17.5 (n = 4)	61% (n = 13)	587
			2	160	13.7 (n = 5)	50% (n = 8)	899
			3	100	12.6 (n = 6)	62% (n = 60)	2048
			4	160	40.2 (n = 6)	75% (n = 60)	598
						Mean ± SD	1033 ± 692
	EEEV	0.09	1	630	27.3 (n = 5)	54% (n = 31)	56
			2	4000	3.3 (n = 5)	16% (n = 23)	33
						Mean ± SD	44 ± 16
*Ae*. *aegypti*	VEEV	0.47	1	80	77.9 (n = 5)	11% (n = 30)	174
	EEEV	0.10	1	400	111.9 (n = 5)	10% (n = 32)	9

*Number of plaque-forming units (PFU) of virus needed to infect 50% of the mosquitoes when injected into their thoraces.

Raw data and dose-response equations are presented in S1 Table.

## Materials and methods

### Viruses, mosquitoes, microfilariae, and gerbils

Two viruses were used; VEEV, V3000 infectious clone of the epizootic subtype 1A Trinidad donkey strain [13] and EEEV, FL91-4679 strain isolated from *Ae*. *albopictus* collected in Polk County, Florida USA [14]. The VEEV V3000 clone was supplied by Dr. J Smith (U.S. Army Medical Research Institute of Infectious Diseases) and the EEEV FL91-4679 strain was supplied by the Centers of Disease Prevention and Control. The precise histories of the strains are not known but both were considered ‘low-passage’ strains, having not been passaged in cell culture more than three or four times prior to use in these experiments. Infectious virus was quantified by plaque assay on Vero cell monolayers as described by Gargan and other [15], except that the second agar overlay containing neutral red was added 2 days rather than 4 days later. Two species of long-colonized *Aedes* mosquitoes were used; *Ae*. *aegypti* Rockefeller strain and *Ae*. *taeniorhynchus* Vero Beach strain. This study used seven male mongolian gerbils (*Meriones unguiculatus*) that were 7–12 weeks old. Microfilaremic gerbils, interperitoneally infected with *Brugia malayi* filarial parasites, were provided by the NIH/NIAID Filariasis Research Reagent Resource Center for distribution by BEI Resources, NIAID, NIH (NR-49238). Nonmicrofilaremic gerbils were obtained from Tumblebrook Farm, Brookfield, MA USA. Gerbils were maintained in standardized rat cages fitted with filter tops and supplied wood-chip bedding and enrichment toys (plastic tubes and balls). Gerbils were maintained on standard rat chow, room temperature and light cycle and were monitored twice daily by a trained animal care technician throughout the experiment. An institutionally approved protocol was in place that defined the early/humane endpoint and in the event that animals became visibly ill (hunched back, ruffled fur, etc.) gerbils were to be euthanized via CO_2_ inhalation. None of the animals became ill or died prior to the experimental endpoint. Gerbils were anesthetized with an approved anesthetic (intraperitoneal injection of ketamine-acepromazine-xylazine mixture) prior to having mosquitoes feed on them. After the experiment, gerbils were euthanized via CO_2_ inhalation.

### Innate susceptibilities of mosquitoes to virus infection

To compare relative susceptibilities to hemocoelomically introduced virus, 4- to 10-day-old nulliparous female mosquitoes were inoculated [16] with VEEV or EEEV. Briefly, serial dilutions were made from virus stock solutions of known concentration, expressed as plaque-forming units per ml (PFU/ml). Mosquitoes were chilled and handled on a chill table to facilitate the procedure. Each mosquito (10 to 25 per dose) received an intrathoracic injection of 0.3 μl virus suspension delivered with a fine-tipped glass capillary needle that had been previously calibrated, marked in 0.1-μl increments and powered by positive displacement (compressed air). Injected mosquitoes were placed in recovery cages and held at 26°C for 7 days at which time mosquitoes were homogenized individually by hand in glass grinders containing 1 ml of diluent. Mosquito homogenates were assayed for infectious virus by plaque assay [15].

### Quantitative estimates of viremic blood introduced per MF

As mentioned previously, our studies with VEEV and *B*. *malayi* yielded higher rates of viral dissemination that did studies with EEEV and *B*. *malayi*, despite the fact that host viremias were higher with EEEV. To investigate further these unanticipated findings, calculations were made to estimate the proportional amount of viremic blood ingested by a dually-infected mosquito that would have to have been introduced into the hemocoel by each penetrating MF in order to yield the level of enhancement observed in our trials. We call this estimate ‘*parts per blood meal’* (ppBM) per MF. For each trial, the host viremia and numbers of penetrating MF had been recorded and thus we used these data, in combination with the dose-response equations generated from inoculation studies described above, to calculate ppBM estimates for each VEEV and EEEV trial. For example in a trial wherein *Ae*. *taeniorhynchus* were fed on a dually-infected gerbil, there was a 54% net increase in the EEEV dissemination rate over that in the control group. Substituting 0.54 for the Y-value in the dose-response equation for relative susceptibility of *Ae*. *taeniorhynchus* to inoculated EEEV and solving for the X-value, it was calculated that a hemocoelomically delivered dose of 0.096 PFU of EEEV virus per mosquito was required to infect 54% of the mosquitoes. The average viremic content of the actual blood meals was measured at 630 PFU per mosquito and the geometric mean number of penetrating MF per mosquito was 27.3. Therefore, the total proportion of viremic blood meal that must have been introduced into the hemocoel per MF to produce a 54% enhanced dissemination rate was: (0.096 PFU / 630 PFU) ÷ 27.3 MF or 0.0000056 parts of the blood meal per MF (ppBM). In this way we calculated ppBM values for each trial in which there occurred a significant enhancement of EEEV or VEEV dissemination due to concurrent ingestion of *B*. *malayi* MF by mosquitoes.

### Kinetics of viral growth

The kinetics of viral growth within the hemocoels of infected mosquitoes were compared between mosquitoes fed on dually-infected gerbils [9] versus mosquitoes infected by intrathoracic inoculation. Immediately after virus exposure, batches of 10 to 30 mosquitoes from each group were placed individually in appropriately labeled cages. Over the course of several days, mosquitoes were immobilized by chilling and a single leg was carefully amputated at the coxa/trochanter joint from each mosquito, after which it was returned to its cage. The order in which legs were removed (e.g., right front leg, right rear leg, left middle leg) permitted mosquitoes to maintain a tripod for stability and thus retain the ability to stand, walk, and stay alive for the duration of the sampling period. For mosquitoes fed on dually-infected gerbils, legs were removed on days 2, 3, and 4 after infection. For inoculated mosquitoes, legs were removed on days 1, 2, and 3 after infection. Individual legs were ground in 1 ml culture media using glass tissue grinders and labeled as to the mosquitoes they belonged. On the last day (i.e., day 4 for inoculated mosquitoes, day 5 for blood-fed mosquitoes), the remaining three legs were amputated and legs and bodies were ground separately. Virus within samples was quantified using plaque assays.

### “Spin and wash” experiments

Several thousand *Brugia malayi* MF were incubated for one hour at 35°C in 5 ml of cell culture medium to which was added either VEEV (starting titer = 10^5.6^ PFU/ml) or EEEV (starting titer = 10^3.5^ PFU/ml). Tubes were gently agitated every 5–10 minutes to ensure mixing of MF and virus. Afterwards, tubes were centrifuged for 5 minutes at 1,000 rpm to pellet the MF and 4.5 ml of the supernatant was removed and stored at -70°C for later viral quantification. The MF pellets were re-suspended by adding 4.5 ml of fresh medium. Tubes were vortexed for several minutes to ensure mixing and then centrifuged again at 1,000 rpm to pellet the MF. This procedure of washing the MF with serial 10-fold dilutions was repeated eight times in order to dilute virus beyond the theoretical limits of detection. After the final centrifugation and supernatant removal, the residual 0.5 ml of diluent containing the MF pellet was vortexed and the MF ‘slurry’ was assayed for virus by plaque assay. A small amount (ca. 100 μl) was reserved to inoculate into the thoraces of *Ae*. *aegypti* mosquitoes (3 μl per mosquito) as described above. Inoculated mosquitoes were maintained for 7 days and then homogenized individually and assayed for virus by plaque assay.

### Data analyses

Rates of viral infection and dissemination were compared among groups by chi square analyses or Fisher’s exact test, depending on sample size. To construct dose response equations, linear regression analyses were performed with viral dose transformed to log_10_ as the independent variable and infection rates transformed to probits as the dependent variable. The software package, Statistix (Tallahassee, FL) was used with the 0.05 level of significance throughout.

### Ethics statement

Research at U.S. Army Medical Research Institute of Infectious Diseases (USAMRIID) was conducted under an Institutional Animal Care and Use Committee (IACUC) approved protocol in compliance with the Animal Welfare Act, PHS Policy, and other federal statutes and regulations relating to animals and experiments involving animals. This facility where this research was conducted is accredited by the Association for Assessment and Accreditation of Laboratory Animal Care, International and adheres to the principles stated in the *Guide for the Care and Use of Laboratory Animals*, National Research Council, 2011. The USAMRIID IACUC specifically approved this study.

## Results

### Quantity of viremic blood introduced per MF

In *Ae*. *taeniorhynchus*, the mean ppBM for VEEV introduced into the hemocoel per MF (1,033 x 10^−7^ ppBM; n = 4) was over 23 times greater than that estimated for EEEV (44 x 10^−7^ ppBM; n = 2) (Table 1). Similarly, in *Ae*. *aegypti*, the ppBM for VEEV (174 x 10^−7^ ppBM; n = 1) was 19 times greater than that estimated for EEEV (9 x 10^−7^ ppBM; n = 1) (Table 1). Replicates were too few for differences to be statistically significant. Nevertheless, if MF-mediated viral dissemination were due solely to the amount of blood leaking out from punctures in the midgut, one would expect ppBM values within each mosquito/MF species combination to be roughly equivalent, regardless of the virus. Instead, the differences in these estimates suggested that the amount of virus introduced into the hemocoel by MF depended not only on the MF/mosquito species combination because of differences in blood meal leakage, but also upon the virus being examined. Passive leakage of virus into the hemocoel by itself was insufficient to explain these observations.

### Growth kinetics of disseminated virus

**Fig 2:**
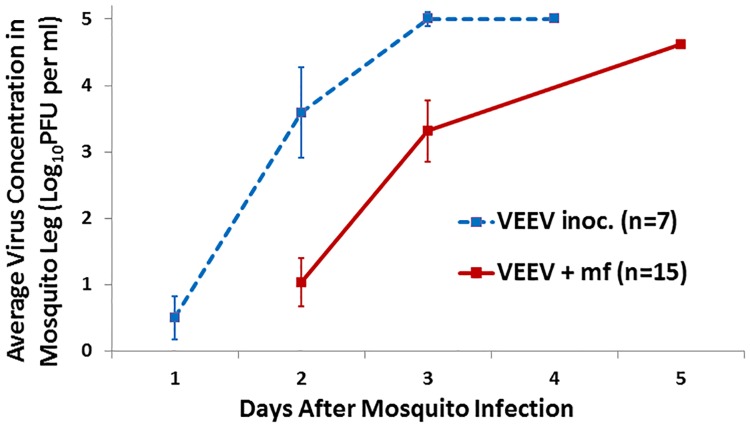
Comparative growth kinetics of Venezuelan equine encephalitis virus in *Aedes taeniorhynchus* mosquitoes when virus is introduced into the hemocoel *via* direct inoculation (VEE inoc.) versus *Brugia malayi* microfilarial passage through the midgut (VEE + mf).

Growth kinetics of VEEV within the hemocoels (i.e., amputated legs) of *Ae*. *taeniorhynchus* mosquitoes were compared between mosquitoes whereby virus was introduced by MF (= virus + MF) versus direct inoculation into the thorax. Mosquitoes tolerated serial amputations well, with over 95% survival at the end of the 5-day trial (Table 2). It took an average of 1.9 ± 0.7 days for virus to first be detected in the legs of intrathoracically-inoculated mosquitoes (n = 7) whereas it took 3.0 ± 1.1 days for virus to first be detected in the legs of mosquitoes fed on a dually-infected host (n = 15) (t = -2.44, df = 20, p = 0.024). Thus, there was a delay of ca. 1 day in the detection of virus and nearly a 2-day delay in reaching peak titers in mosquitoes infected *per os* on dually-infected blood (virus+MF) versus mosquitoes infected by intrathoracic inoculation (Fig 2). Importantly, the estimated quantities of virus delivered by MF versus inoculation were equivalent (0.3 PFU per mosquito, Table 2). Furthermore, the inoculated mosquitoes received essentially the minimal amount of infectious virus needed to cause an infection (i.e., 1 ID_50_ = 0.2 PFU, Table 1). Therefore, the resultant growth curve (Fig 2) represents the slowest possible kinetics and most protracted lag phase for VEEV growth that can occur within *Ae*. *taeniorhynchus* when virus is injected directly into the hemocoel and mosquitoes are subsequently maintained under typical insectary conditions. If VEEV had also been available for replication within the hemocoel shortly after mosquitoes had ingested VEEV plus MF (i.e., if virus had simply leaked out the holes made by the MF), then the growth curves for the two sets of mosquitoes (inoculated versus *per os*) should have been identical. However, if the virus was not immediately available, then there would have been a delay in the growth curve. This is what was observed (Fig 2).

**Table 2:** Mosquito survival, comparative infection rates, and estimated amount of virus delivered into the hemocoel of mosquitoes during trials comparing the kinetics of viral dissemination in *Aedes taeniorhynchus* mosquitoes infected *per os* on a dually-infected gerbil with concurrent Venezuelan equine encephalitis virus viremia and *Brugia malayi* microfilaremia (VEEV + mf) versus mosquitoes injected with Venezuelan equine encephalitis virus directly into the thorax (VEEV inoc.).

	VEEV + mf	VEEV inoc.
Number of mosquitoes at beginning of trial	28	10
Number of mosquitoes at end of trial (% survival)	27 (96%)	10 (100%)
Number of virus-infected mosquitoes at end of trial (% infected)	22 (81%)	7 (70%)
Number of disseminated viral infections at end of trial (% disseminated)	15 (68%)	7 (70%)
Estimated amount of VEEV delivered into the hemocoel *	0.31 PFU	0.33 PFU

* Calculated from dose-response equation (See S1 Table).

### Spin-and-wash experiments

The affinity of the MF/virus association was examined by incubating MF with virus, then subjecting the MF to repeated cycles of washing and then assaying the washed MF pellet for virus. In the EEEV trial, the starting virus titer was 10^3.5^ PFU/ml and titers of the supernatants decreased logarithmically with each successive wash until the fourth wash, at which point there was no detectable virus left in successive supernatants (Table 3). For the VEEV trial, the starting virus titer was much higher (10^5.6^ PFU/ml). During the dilution series, virus titers in the supernatants leveled off at 10^2.3^ PFU/ml after the fifth wash and did not diminish with successive washings. After the final wash, MF pellets for both viruses were assayed by plaque assay and by mosquito inoculation. No EEEV was detected in the MF pellet by plaque assay. Examination of the plaque assay wells with an inverted microscope revealed an abundance of pelleted MF trapped within the solidified agar overlay, but no virus plaques were present. However, nearly half of the 24 *Ae*. *aegypti* mosquitoes inoculated with 0.3 μL of the pelleted MF suspension developed viral infections 1 week after inoculation. This indicated that residual EEEV had remained in or on the MF even though it was undetectable by direct plaque assay. Mosquito inoculations were more sensitive than the plaque assays perhaps because once inoculated, infective virions had an additional week in which to replicate within the mosquito hemocoel before being processed for plaque assay. In the VEEV trial, virus was detected at high levels both by plaque assay and mosquito inoculation. Pelleted MF within the agar of the plaque assays were surrounded by large coalescing plaques and all 24 inoculated mosquitoes became infected. These results suggest that both EEEV and VEEV became associated with *B*. *malayi* MF during the hour-long incubation and remained in or on the MF despite repeated washings. The higher viral concentration of VEEV in the MF pellet, as determined by both plaque assay and mosquito inoculation, suggests that viral affinity to *B*. *malayi* MF was higher with VEEV than with EEEV.

**Table 3:** Detection of virus associated with *Brugia malayi* microfilariae (MF) incubated for 1 hour with either Venezuelan equine encephalitis (VEEV) or eastern equine encephalitis (EEEV) viruses and then extensively washed by repeated cycles of centrifugation, removal of supernatant and resuspension. Viruses were detected in the final MF pellet by standard plaque assay and via inoculation into *Aedes aegypti* mosquitoes.

Virus	--------- Virus titer (log_10_ PFU per ml) of supernatant at each dilution ---------									Virus titer of MF pellet (log_10_ PFU per ml)	Infection of mosquitoes inoculated with MF pellet
	Stock	10^−1^	10^−2^	10^−3^	10^−4^	10^−5^	10^−6^	10^−7^	10^−8^		
EEEV + MF	3.5	2.4	1.5	1.0	0	0	0	0	0	0	11 / 24
VEEV + MF	5.6	4.6	3.5	2.7	2.6	2.3	2.3	2.3	2.3	4.4	24 / 24

## Discussion

**Fig 3:**
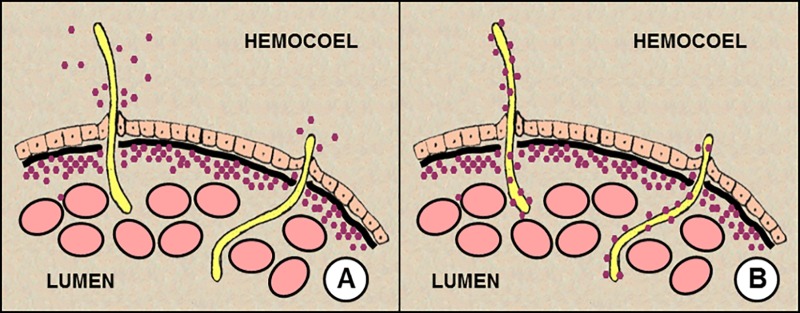
Revised mechanisms of microfilarial enhancement of arboviral transmission. **A**. Virus leaks from the exit hole made by penetrating mf. **B**. Virus adheres to or is somehow associated with microfilariae in the blood and is transported across the midgut by microfilariae during the process of penetration. The relative importance of either mechanism may depend on the species combination of mosquito, microfilaria, and virus.

Experimental evidence indicated that VEEV and EEEV were actively transported into the mosquito hemocoel by *B*. *malayi* MF during their exodus from the midgut. This is different than the simple leakage of viremic blood into the hemocoel from holes or lacerations caused by penetrating MF. When mosquitoes feed on a host that is concurrently viremic and microfilaremic, both pathogens are ingested and infectious virions may enter the mosquito hemocoel and establish a disseminated viral infection sooner than normal [6, 10]. This is the basis of MF enhancement of arboviral transmission. The simplest explanation for this has been that a small amount of the infectious blood meal is introduced into the hemocoel as a result of leakage from puncture sites made by MF as they pass through the midgut. Presumably, the more MF that penetrate the midgut (i.e., high microfilaremia) and the more extensive is the damage done to the midgut (e.g., large MF), the more likely it is that virus would be introduced. Similarly, the more virus there is in the bloodmeal (i.e., high viremia), the more likely that virus would be introduced. In this report, we present several lines of evidence to suggest that active conveyance of virus into the hemocoel by MF, rather than leakage of viremic blood, is the mechanism responsible for MF enhancement of VEEV and EEEV by *B*. *malayi* MF (Fig 3).

First, the estimated amount of viremic blood introduced per *B*. *malayi* MF into the hemocoel of *Ae*. *taniorhynchus* mosquitoes was much greater for VEEV (1,033 x 10^−7^ ppBM) than for EEEV (44 x 10^−7^ ppBM) (Table 1). These calculations took into account the observed number of penetrating MF for each experimental replicate and thus represent the ‘*per capita*’ amount of blood meal leakage. One would expect experiments with different viruses would yield comparable estimates if the introduction of virus were mediated solely by passive leakage from MF puncture sites. But they did not. This study suggested that the magnitude of transport depended not only on the mosquito/MF species combination, but also on the virus/MF species combination. More VEEV was transported per penetrating MF than was EEEV.

Second, there was a delay of ca. 24 hours in the growth kinetics of virus within mosquito hemocoels when virus was delivered by MF versus by direct inoculation (Fig 2). Studies on the kinetics of *Brugia* spp. MF passage through the mosquito midgut indicate that MF penetration occurs rapidly and passage is largely complete within 3 to 4 hours after an infective meal [17–21]. Therefore if virus introduction into the hemocoel were mediated solely by leakage from MF puncture sites, one would have expected that the pattern of viral growth kinetics would have been more similar to that of inoculated virus. It was not.

Third, MF were incubated with virus and then subjected to a series of repeated washings so extensive that any residual virus should have been diluted beyond the theoretical limits of detection. Yet the washed MF still contained infectious virus (Table 3). This indicated that the virus had become associated in some way with the MF. Furthermore, the magnitude of this association appeared to be greater with VEEV than with EEEV, consistent with our calculations for ppBM (Table 1).

The precise nature of this association remains unknown but one possibility may be related to differences in the electrostatic charges between *Brugia* MF and VEEV and EEEV. Studies whereby *Brugia pahangi* MF were incubated in a suspension of cationic colloidal iron demonstrated that the microfilarial sheaths were strongly electronegative. However, once the MF had penetrated the mosquito midgut and shed their sheaths, the surfaces of MF became electro-neutral [22]. Most studies have shown that *Brugia* MF retain their sheaths following ingestion by *Aedes* spp. mosquitoes and that sheaths are only shed after MF pass through the midgut [12, 17–18, 20, 23–25]. However, a few studies have shown that in certain strains of *Ae*. *aegypti*, exsheathment of ingested MF may occur prior to midgut penetration [17, 26]. The degree to which MF exsheath before or after midgut penetration within a particular mosquito species or strain may affect the magnitude of MF enhancement that occurs and could explain the species differences observed in our earlier studies (Table 1). In our experiments, the Trinidad Donkey strain of VEEV and the FL91-4679 strain of EEEV were used. Both of these strains of alphaviruses have been shown to possess a positively-charged surface glycoprotein, E2 protein, which is believed to serve as a major attachment ligand, binding to electro-negative heparin sulfate moieties on vertebrate host cells [27–29]. If the associations between *B*. *malayi* MF and EEEV/VEEV were mediated by charge interactions between the MF sheaths (= negative) and virus (= positive), and the sheaths were retained until after MF crossed the mosquito midgut, this would explain the 24-hour delay in viral growth kinetics between MF-mediated versus direct inoculation modes of viral entry into the mosquito hemocoel. Perhaps during the host viremia, virus adheres to the sheath of MF within the blood of a dually-infected host but disassociates only after MF pass through the midgut and the virus-coated sheaths are shed within the mosquito hemocoel.

In areas where arboviral and filarial infections co-occur and prevalence of filariasis is high, MF enhancement may act to intensify natural arboviral transmission cycles. However, MF enhancement can only occur if the following 5 conditions are met. First, a vertebrate host must be concurrently infected with both virus and microfilariae. Second, mosquitoes feeding on a dually infected host must ingest both virus and microfilariae. Third, microfilariae must penetrate the mosquito midgut. Fourth, upon microfilarial penetration, sufficient virus must pass into the mosquito body cavity to establish infection. Fifth, there must be no salivary gland barriers and the mosquito must be able to transmit the virus by bite. In this report, we have identified a complexity to the fourth condition. That is, potential introduction of virus into the hemocoel is dependent not only on the host microfilaremia (i.e. number of punctures caused by MF), viremia (i.e. dose of inoculum), and the amount of tissue damage produced during MF penetration (i.e. size of punctures) but also—and perhaps more importantly—on the affinity of virus to adhere to or otherwise associate with the MF. If the virus-MF affinity is weak or nil, then virus introduction into the hemocoel could only result from leakage from punctures. Relatively high levels of microfilaremia, viremia, and/or expansive midgut fissures created during MF penetration would be required to produce enhanced viral dissemination in the vector. The degree to which MF typically create midgut fissures remains unclear. Some histological studies on MF penetration have described lesions disrupting the full depth of the midgut wall [23–24] whereas other studies describe MF taking a more circuitous route, moving intracellularly across adjacent midgut epithelial cells and producing little pathology before exiting [30]. At the viremia tested in our kinetics study with VEEV and *Ae*. *taeniorhynchus*, we did not find evidence of virus leakage into the hemocoel. However, at higher viremias it is entirely possible that infectious virus might leak out from the exit sites created by MF in sufficient quantities to produce enhanced dissemination.

If on the other hand, the affinity between virus and MF is strong (as observed for VEEV and *Brugia*), then MF enhancement may occur at lower host microfilaremia and/or viremia, and require fewer, less destructive MF penetrations through the midgut. In this regard, it is important to note that some MF species are sheathed whereas others are not. The presence or absence of a sheath, as well as the physio-chemical composition of the sheath, may affect virus-MF affinity and thus influence the probability of MF enhancement occurring.

Microfilarial infections in wildlife are commonplace [31–39]. Animals that are simultaneously microfilaremic and seropositive to arboviruses have been documented in the wild—e.g. *Mansonella*/*Dipetalonema* filariases and Mayaro virus in neotropical monkeys [40], *Chandlerella*/*Eufilaria* filariases and West Nile virus in North American songbirds [41]. This indicates that wildlife can harbor both filarial and arbovirus infections concurrently. There may be many zoonotic arboviral transmission systems where MF enhancement could potentially be involved. Our results suggest that in each system, the interacting components of vector, parasite and virus will have their own unique characteristics that determine the likelihood of MF enhancement occurring. Thus, MF enhancement may be important in one transmission system but unimportant in another. This will depend on how well the five previously mentioned conditions are met, as well as the affinity of virus to associate with MF during a viremia within the blood of microfilaremic host.

## Supporting information

S1 Table Dose-response of *Aedes* spp. mosquitoes to intrathoracically inoculated Venezuelan equine encephalitis virus (VEEV) and eastern equine encephalitis virus (EEEV). (DOCX)
