# Supplementary material for: Brugia malayi microfilariae transport alphaviruses across the mosquito midgut
Source: PLoS One. 2017 Feb 21;12(2):e0172309. doi: 10.1371/journal.pone.0172309 (PMC5319744; doi:10.1371/journal.pone.0172309)
Supplement: S1 Table — (DOCX) [file pone.0172309.s001.docx]

**S1 Table**. Dose-response of *Aedes* spp. mosquitoes to intrathoracically inoculated Venezuelan equine encephalitis virus (VEEV) and eastern equine encephalitis virus (EEEV).

|  |  |  | *Aedes taeniorhynchus* | | *Aedes aegypti* | |
| --- | --- | --- | --- | --- | --- | --- |
| Virus | Dose (PFU/ml) | Log_10_ (Dose) | Percent Infected | Probit Percent Infected | Percent Infected | Probit Percent Infected |
| VEEV | 0.05 | -1.30 | 20.0% (n=5) | 4.16 | 11.5% (n=26) | 3.80 |
|  | 0.10 | -1.00 | 37.5% (n=16) | 4.68 | 4.8% (n=21) | 3.35 |
|  | 0.20 | -0.70 | 21.7% (n=23) | 4.21 | 12.5% (n=24) | 3.84 |
|  | 0.40 | -0.40 | 57.9% (n=19) | 5.20 | 26.9% (n=26) | 4.39 |
|  | 0.80 | -0.10 | 81.2% (n=16) | 5.89 | 79.0% (n=19) | 5.81 |
|  | 1.60 | 0.20 | 100.0% (n=5) | 8.09 | 90.0% (n=10) | 6.28 |
| Dose-Response Equation | | | Probit Percent Infected = (2.3035)(log_10_Dose) + 6.6350 | | Probit Percent Infected = (1.9296)(log_10_Dose) + 5.6366 | |
| EEEV | 0.03 | -1.51 | 10.0% (n=10) | 3.72 | 0% (n=10) | - |
|  | 0.06 | -1.21 | 14.0% (n=14) | 3.92 | 40.0% (n=10) | 4.75 |
|  | 0.12 | -0.90 | 60.0% (n=10) | 5.25 | 60.0% (n=10) | 5.25 |
|  | 0.25 | -0.60 | 100.0% (n=9) | 8.09 | 60.0% (n=10) | 5.25 |
|  | 0.50 | -0.30 | 90.0% (n=10) | 6.28 | 100.0% (n=10) | 8.09 |
| Dose-Response Equation | | | Probit Percent Infected = (3.0805)(log_10_Dose) + 8.2403 | | Probit Percent Infected = (3.3237)(log_10_Dose) + 8.3418 | |
